# Concurrent *BCR‐ABL1* and core binding factor beta rearrangement in de novo acute myeloid leukemia: A case report and review of literature

**DOI:** 10.1002/jha2.895

**Published:** 2024-04-17

**Authors:** Brittany Salter, Sarah Ge, Amy Tam, Suzanne Demczuk, Darci Butcher, Elizabeth McCready, Dina Khalaf

**Affiliations:** ^1^ Department of Medicine McMaster University Hamilton Canada; ^2^ Department of Hematology Grand River Hospital Kitchener Canada; ^3^ Department of Pathology and Molecular Medicine McMaster University Hamilton Canada; ^4^ Genetics Laboratory, Hamilton Regional Laboratory Medicine Program Hamilton Health Sciences Hamilton Canada; ^5^ Department of Oncology McMaster University Hamilton Canada

**Keywords:** AML, BCR‐ABL1, case report, CBFB rearrangement

## Abstract

A distinct subset of acute myeloid leukemia (AML) is characterized by the presence of the Philadelphia chromosome (Ph+), due to reciprocal translocation t(9;22)(q34;q11.2). This chromosomal rearrangement leads to the fusion of the breakpoint cluster region (BCR) gene on chromosome 22 with the ABL1 gene on chromosome 9, generating the *BCR::ABL1* fusion gene. The Ph+ AML subtype is associated with poor prognosis and resistance to conventional chemotherapy. Beyond the well‐established *BCR::ABL1* fusion, recent studies have shed light on additional genetic abnormalities in Ph+ AML, including associations with rearrangements involving core binding factor beta (CBFB). We describe a case of de novo AML with concurrent *BCR::ABL1* and *CBFB::MYH11* rearrangements.

## 1 INTRODUCTION

The Philadelphia chromosome (Ph) is the product of a reciprocal translocation between the long arms of chromosomes 9 and 22, t(9;22)(q34;q11.2). This rearrangement brings together the *BCR* gene on chromosome 22 and the *ABL1* gene on chromosome 9, resulting in the formation of the *BCR::ABL1* fusion gene. The *BCR::ABL1* fusion protein, a constitutively active tyrosine kinase, drives the dysregulated proliferation and impaired differentiation of myeloid cells. The *BCR::ABL1* fusion gene is known for its association with chronic myelogenous leukemia (CML) [1] but has also recently become its own entity in de novo acute myeloid leukemia (AML) [2].

Chromosomal rearrangements involving the core binding factor (CBF), including t(8;21)/*RUNX1*::*RUNX1T1* or inv(16)/t(16;16)/*CBFB*::*MYH11*, are classified as AML‐defining alterations [2]. The inv(16) results in the fusion of the myosin heavy chain 11 gene (*MYH11*) at 16p13 and the core binding factor beta subunit gene (*CBFB*) at 16p12. This, in turn, disrupts normal hematopoietic regulation and contributes to leukemogenesis. CBF‐related chromosomal abnormalities are associated with favorable prognosis in AML [3, 4].

The co‐occurrence of *CBFB* and *BCR‐ABL1* rearrangements in AML represents a distinct subgroup with unique clinical and molecular features. To date, there have been less than 30 cases reported in the literature [1, 5–24]. This intriguing association, while rare, poses a unique diagnostic and therapeutic challenge, as the treatment strategies for CML and AML markedly differ. We present a clinical case of de novo AML with concurrent *BCR‐ABL1* and *CBFB‐MYH11* rearrangements and discuss the diagnostic challenges, treatment implications, and prognostic implications.

### 1.1 CASE PRESENTATION

**FIGURE 1:**
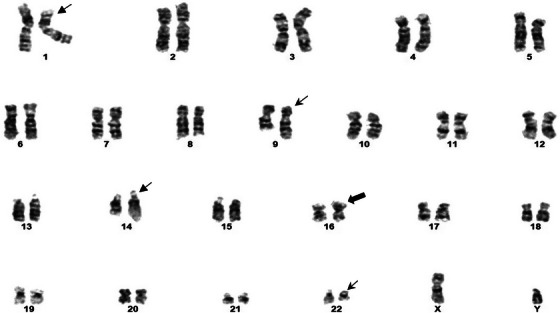
Cytogenetic bone marrow test at initial diagnosis, The cytogenetic bone marrow revealed an abnormal karyotype with three distinct abnormalities including a balanced reciprocal translocation between the short arm of one chromosome 1, arrow, and long arm of one chromosome 14, with breakpoints within bands 1p32 and 14q24, reciprocal translocation between long arms of chromosomes 9 and 22, arrows, with breakpoints at bands 9q34 and 22q11.2, pericentric inversion of chromosome 16, with breakpoints at bands 16p13.1 and 16q22.

A 62‐year‐old male presented to the hospital with headaches, diarrhea, and weakness. He had a past medical history of diabetes and dyslipidemia, with no personal or family history of malignancy or chemoradiation exposure. Testing revealed leukocytosis (24.1 × 10^9^/L), anemia (hemoglobin 110 g/L), thrombocytopenia (platelets 98 × 10^9^/L), neutropenia (0.8 × 10^9^/L), and circulating blasts (16.2 × 10^9^/L; 42.5%). The bone marrow (BM) was hypercellular (90%) with 56% myeloblasts, consistent with AML (non‐M4 Eosinophilia French American‐British System). Flow cytometry revealed a population of myeloblasts (CD34+, partial HLA‐DR+, CD117+, CD33+, CD13+, and cytoplasmic MPO+) that were 34% of the total leukocyte population. Cytogenetic testing revealed an abnormal karyotype in all ten metaphases examined, with three chromosomal rearrangements (Figure 1). The first is a balanced reciprocal translocation between the short arm of chromosome 1 and the long arm of chromosome 14 with breakpoints at bands 1p32 and 14q24. The second was a reciprocal translocation between the long arms of chromosomes 9 and 22 with breakpoints at bands 9q34 and 22q11.2 [t(9;22)]. The third was recurrent pericentric inversion of chromosome 16 with breakpoints at bands 16p13.1 and 16q22. A qualitative reverse transcriptase polymerase chain reaction (RT‐PCR) detected a transcript that was consistent with a *BCR::ABL1* fusion gene having an e19a breakpoint, encoding a 220 kDa fusion protein product (p230). These findings were consistent with concurrent *BCR::ABL1* and *CBFB::MYH11* fusion gene transcripts.

The patient was treated with 7+3 induction (standard dose cytarabine 100 mg/m^2^ for 7 days; daunorubicin 60 mg/m^2^ for 3 days). The detection of *BCR::ABL1* fusion transcript prompted the initiation of the tyrosine kinase inhibitor (TKI), dasatinib (70 mg po BID), to his regimen from Day 8 onwards. Given evidence of *CBFB::MYH11* fusion transcript and CD33+ myeloblasts, the use of gemtuzumab ozogamicin (GO) was offered, but the patient refused.

His hospital stay was complicated by grade two mucositis and febrile neutropenia, secondary to PICC line‐associated infection with *enterococcus faceium* bacteremia, and treated with a two‐week course of vancomycin. He developed vancomycin‐induced nephrotoxicity, with a peak creatinine of 325 micromol/L. He also developed facial edema and diffuse morbilliform rash, thought to be related to dasatinib, which was held on Day 22. A skin biopsy showed non‐specific perivascular inflammation. The rash improved with dasatinib discontinuation, which was restarted on Day 37 with no issue.

Count recovery was evident on Day 19 with hemoglobin 74 g/L, platelets 106 × 10^9^/L, and neutrophils 1.2 × 10^9^/L. A BM done on Day 33 showed morphologic remission with no detection of *BCR::ABL1* or *CBFB::MYH11* fusion gene transcripts via nested RT‐PCR. Consolidation included infusional cytarabine with renal dosing at 50 mg/m^2^ for 7 days. The consolidation course was complicated by febrile neutropenia, severe deconditioning, and poor mobility, with a stage two sacral ulcer, worsening renal impairment, and transaminitis, for which dasatinib was held for 7 days. A BM examination was repeated on Day 76 and demonstrated continued morphologic and cytogenetic remission. His poor performance status and comorbidities precluded him from an allogeneic stem cell transplant (allo‐SCT) or further intensive chemotherapy. As such, he received maintenance therapy with dasatinib and oral azacytidine (300 mg daily, Days 1–14, every 28 days). At 6‐month follow‐up, the patient remains alive and well.

## 2 DISCUSSION

Ph+ AML is rare, ranging from 0.5% to 3%, and is associated with poor prognosis and resistance to standard AML therapies [2, 9, 10]. There have been few cases with Ph+ AML that incorporate additional genetic abnormalities, including inv(16), t(8;21)/*RUNX1::RUNX1T1*, t(15;17)/*PML::RARA*, inv(3), 5q deletion, and *NPM1* mutations [11 ^,^ 12]. Based on our literature search, less than 30 cases have been reported regarding concurrent *BCR‐ABL1* and *CBFB‐MYH11* rearrangements in leukemia (Table 1). The majority of cases are in the CML‐blast phase (CML‐BP), where the progression from chronic to BP is thought to be secondary to the sequential acquisition of inv(16)(p13q22) on top of a pre‐existing *BCR::ABL1* rearrangement [1, 25]. The second patient group consists of de novo AML, where genetic alterations of *BCR::ABL1* and *CBFB::MYH11* were discovered simultaneously [1].

**TABLE 1:** Summary of patient clinical features across case reports of de novo acute myeloid leukemia (AML).

Reference and Year	Patient	Age at initial dx	Sex (M/F)	Therapy	AlloSCT (Yes/No)	Response to therapy	Status at last FU	Time to relapse (months)	Time from diagnosis to last FU (months)
Salem et al. 2017 [1]	1	71	M	FLAG‐Ida Dasatinib	No	Remission	Alive	N/A	21
Salem et al. 2017 [1]	2	66	M	Induction with 7+3 Consolidation with high dose cytarabine; MEK inhibitor GSK1120212; single agent decitabine	No	Relapse	Dead	8	24
Salem et al. 2017 [1]	3	55	F	High dose cytarabine, idarubicin; imatinib+clofarabine and cytarabine; dasatinib	No	Relapse	Dead	7	11
Sethapati et al. 2020 [5]	4	55	M	Gemtuzumab ozogamicin induction + Dasatinib	No	Relapse	Dead	6	8
Secker‐Walker et al. 1992 [6]	5	40	M	Standard chemotherapy (Unknown)	Yes	Remission	Alive	N/A	18
Secker‐Walker et al. 1992 [6]	6	9	F	MRC trial UKAML 10	Yes	Remission	Alive	N/A	Unknown
Svaldi et al. 2001 [7]	7	25	M	7+3 Relapse: DAT and COAP, intrathecal Ara‐C, methotrexate	No	Relapse	Dead	3	15
Zhang et al. 2019 [8]	8	30	F	Imatinib, hydroxyurea, busulfan, and cyclophosphamide	Yes	Relapse	Dead	29	81
Zhang et al. 2019 [8]	9	35	M	High dose cytarabine, imatinib	No	Remission	Alive	N/A	17
Preudhomme et al. 1992 [9]	10	64	M	Daunorubicin‐mitoxantrone, Ara‐c + intensification with etoposide, mitoxantrone, and high dose Ara‐c	No	Remission	Alive	N/A	12
Siddiqui et al. 2002 [10]	11	23	M	Ara‐c, mitoxantrone, etoposide, ATRA induction chemotherapy followed by cytarabine consolidation	No	Remission	Alive	N/A	36
Li et al. 1988 [11]	12	39	M	DAT chemotherapy; relapse also responsive to DAT	No	Remission	Alive	38	65
Wu et al. 2006 [12]	13	44	M	Standard chemotherapy (Unknown)	Yes	Remission	Dead	N/A	24
Miura et al. 1994 [13]	14	40	M	Standard chemotherapy (Unknown)	Yes	Remission	Alive	N/A	27
Tirado et al. 2010 [14]	15	13	M	High dose cytarabine; daunomycin, etoposide, intrathecal cytarabine + gemtuzumab	No	Remission	Alive	N/A	10
Cividin et al. 2004 [15]	16	38	F	Idarubicin and cytarabine followed by imatinib (600 mg/day); relapse treated with two courses of cytarabine	No	Remission	Alive	N/A	12
Bacher et al. 2011 [16]	17	63	M	Hydroxycarbamide, daunorubicin/ cytarabine, and imatinib	No	Relapse	Alive	3	13
Bustamante et al. 2012 [17]	18	49	F	Imatinib	No	Remission	Alive	N/A	4
Zhang et al. 2021 [18]	19	50	M	7+3, DAC, HHT, Ara‐C, and etoposide	No	Remission	Alive	N/A	16
Mecucci et al. 1988 [19]	20	64	M	Standard chemotherapy (unknown)	No	Remission	Alive	N/A	7
Levato et al. 2020 [20]	21	57	M	Induction 7+3 with IV cytarabine 100 mg/m^2^ daily followed by daunorubicin 60 mg/m^2^	No	Relapse	Alive	13	42
Han et al. 2014 [21]	22	30	M	Idarubicine, cytosine, arabinoside, and maintenance with imatinib	Yes	Remission	Alive	N/A	15
Dai et al. 2012 [22]	23	31	M	Hydroxyurea, IA regimen with idarubicin, cytosine arabinoside, and maintenance with imatinib	Yes	Remission	Alive	N/A	22
Vitale et al. 2015 [23]	24	70	M	FLAG‐Ida regimen with fludarabine, cytarabine, idarubicin, filgrastim, and dasatinib	No	Remission	Alive	N/A	5
Roth et al. 2011 [24]	25	30	F	Hydroxyurea, allopurinol, imatinib (600 mg/day), and high‐dose chemotherapy with busulfan and cyclophosphamide	Yes	Relapse	Dead	29	80
Roth et al. 2011 [24]	26	35	M	Induction: anthracycline and cytarabine Consolidation: high dose cytarabine Maintenance: imatinib	No	Remission	Alive	N/A	17
**Our case**	**27**	**62**	**M**	**Induction: 7+3, dasatinib** **Consolidation: Dasatinib and cytarabine** **Maintenance: Dasatinib and azacytidine**	**No**	**Remission**	**Alive**	**N/A**	**8**

Abbreviations: Dx, diagnosis; F, female; FU, follow up; M, male; N/A, not applicable.

Patients with antecedent history of CML with *CBFB::MYH11* and *BCR::ABL1* tend to carry the p210 kD fusion protein [1], whereas de novo AML carries the p190 fusion protein [1]. This dichotomy has been shown by other case reports, suggesting two distinct biological processes in these patient subgroups. As demonstrated in Table 2, the majority of patients with de novo AML have the *BCR::ABL1* p190 fusion protein isoform, with only one case noting the detection of a BCR‐ABL1 p210 fusion signal [22]. To our knowledge, we have reported the only case thus far of concurrent *CBFB::MYH11* and *BCR::ABL1* p230 isoform in de novo AML. The clinical significance of this is unknown and warrants further investigation.

**TABLE 2:** Summary of cytogenetics and molecular findings.

Reference and Year	Patient	Cytogenetics at initial diagnosis (karyotype)	*BCR‐ABL1* fusion protein	Other mutations (Next generation sequencing)
Salem et al. 2017 [1]	1	46,XY,inv(16)(p13.1q22)[3] 46,idem,t(9;22)(q34;q11.2)[17]	p190	None
Salem et al. 2017 [1]	2	46,XY,inv(16)(p13q22)[2] 48,idem,t(9;22)9q34;q11.2),+13,+22[16] 47∼48,idem,+13[cp2[	p190	None
Salem et al. 2017 [1]	3	46,XX,t(9;22)(q34;q11.1),+13,+22[16] 46xXX[1]	p190	None
Sethapati et al. 2020 [5]	4	46,XY,inv(16)(p13.1q22)[2]/46,sl,t(9;22)(q34;q11.2)[20]/46,XX[1].nuc ish(MYH11,CBFB)x3(MYH11 con CBFBx2)[190/200]/(ABL1,BCR)x3(ABL1 con BCRx2)[188/200]	p190	None
Secker‐Walker et al. 1992 [6]	5	46.XY.inv(16)(pl3q22)[17]/46,idem,t(9;22)(q34:qll)	p190	N/D
Secker‐Walker et al. 1992 [6]	6	46,XX,inv(16) (p13q22)(21)/ 46,XX,t(9;22)(q34;q11),inv(16)(p13q22)(8)/46,XX(10)	p190	N/D
Svaldi et al. 2001 [7]	7	46,XX,t(9;22)(q34;q11), inv(16)(p13q22).	N/D	N/D
Zhang et al. 2019 [8]	8	46,XX, t(9;22;17;19)(q34; q11.2; q25; p13.1), inv(16) (p13q22)[19]	p190	None
Zhang et al. 2019 [8]	9	46,XY,der(16)inv(16)(p13q22)del(16)(p11.2p13.1)[2]/46,XY,idem, t(9;22)(q34;q11.2)[18]	p190	None
Preudhomme et al. 1992 [9]	10	46,XY,inv(16)(p13q22), t(9;22)(q34;q11)[30]	M‐BCR	None
Siddiqui et al. 2002 [10]	11	46,XY,t(9;22)(q34;q11.2) inv(16)(p13q22) [all cells analyzed]	N/D	N/D
Li et al. 1988 [11]	12	46,XY(9%)/47,XY,−18,+22, inv(16)(p13q22),del(20) (p12p13),del20(q12q13), t(9;22)(q34;q11),der(16) t(16;?18)(q24;q21), +mar(91%)	N/D	N/D
Wu et al. 2006 [12]	13	46,XY,t(9;22)(q34.1;q11.2) inv(16)(p13.1q22)[19]	p190	N/D
Miura et al. 1994 [13]	14	46,XY,inv(16)(p13q22)[17]/46,idem,t(9;22)(q34;q11)[3]	N/D	N/D
Tirado et al. 2010 [14]	15	46,XY,inv(16)(p13.1q22)[2]/46,idem,del(7)(q22q32)[16]/46,idem,t(9;22;19) (q34;q11.2;p13.1)[2]	N/D	N/D
Cividin et al. 2004 [15]	16	46,XX[22]/46,XX,inv(16) (p13q22)[1]/46,XX,idem, t(9;22)(q34;q11)[25]/46,XX, t(2;9;22)(q32;q34;q11), inv(16)(p13q22)[23]	p190	N/D
Bacher et al. 2011 [16]	17	46,XY,inv(16)(p13q22)[20]/46,XY,t(9;22)(q34;q11),inv(16)(p13q22)[2]	N/D	N/D
Bustamante et al. 2012 [17]	18	46,XX,inv(16)(p13.1q22)[5]/46,XX,t(9;22)(q34;q11.2)[7]/46,XX[8]	p190	N/D
Zhang et al. 2021 [18]	19	46, XY,inv(16)(p13.1q22)[10]	N/D	None
Mecucci et al. 1988 [19]	20	46,XY, t(9;22)(q34:q11.2),inv(16)(p13q22)	N/D	N/D
Levato et al. 2020 [20]	21	46,XY/ 46,XY, inv(16)(p13q22)/46,XY,t(9;22)(q34;q11)	p190	None
Han et al. 2014 [21]	22	46,XY,t(9,22)(q34;q11.2), inv(16)(p13,1q22)[13]/47,idem,+17[15]/48,idem,+8,+17[2]	N/D	N/D
Dai et al. 2012 [22]	23	46,XY,der(8)t(8;10)(p23;q25),der(10)t(8;10)t(10;16)(p13; q22),der(16)inv(16)(p13q22)t(10;16)[4]/46,XY,idem, t(9;22)(q34;q11)[6]	p210	None
Vitale et al. 2015 [23]	24	46,XY,inv(16)(p13.1q22)[3]/46,idem,t(9;22)(q34;q11.2)[17]	p190	None
Roth et al. 2011 [24]	25	46,XX, t(9;22;17;19)(q34; q11.2; q25; p13.1), inv(16)(p13q22)[19]	p190	None
Roth et al. 2011 [24]	26	46,XY,der(16)inv(16)(p13q22)del(16)(p11.2p13.1)[2]/46,XY,idem,t(9;22)(q34;q11.2)[18	p190	None
**Our case**	**27**	**46,XY,t(1;14)(p32;q24),t(9;22)(q34;q11.2),inv(16)(p13.1q22)[10]**	**p230**	**None**

Abbreviation: N/D, not done.

In terms of prognosis, we conducted a literature review to determine patient outcomes in de novo AML with concurrent *CBFB::MYH11* and *BCR::ABL1* rearrangements. Of the 27 cases (including ours), 20 patients (77%) remained alive at the time of the last follow‐up, and among these patients, 19 patients (95%) remained in remission. The median time‐to‐relapse was 8 months (interquartile range [IQR]: 3–38), with a total of seven patients (28.8%) relapsing. The median overall survival (OS) was 16.5 months (IQR: 4–81). In contrast, outcomes in patients with CML‐BP appear to be worse with rapid progression and resistance to chemotherapy [21, 24, 26], and a median OS of 14 months [1]. It appears the concurrent *BCR::ABL1* and *CBFB::MYH11* rearrangements in de novo AML confers a more favorable prognosis with outcomes comparable to that of CBF‐AML. This is in contrast to Ph+AML, which is more aggressive with poor response to traditional AML therapy or TKI alone, with a median survival similar to CML‐BP [21, 27].

The therapeutic management of Ph+ AML patients with dual rearrangements poses significant challenges. The traditional treatment strategies for Ph+ AML, including standard AML chemotherapy regimens, are often ineffective in these cases. The presence of *BCR::ABL1* fusion transcript raises the possibility of utilizing TKIs targeting the BCR‐ABL1 kinase domain, similar to CML management. For CBF‐AML, the anti‐CD33 monoclonal antibody, GO, has shown benefit when added to 7+3 induction therapy for patients with favorable or intermediate‐risk cytogenetics [28]. Based on the 27 case reports to date, there appears to be heterogeneity in chemotherapy regimens for de novo AML with *BCR::ABL1* and *CBFB* rearrangements (Table 1). For example, 7+3 induction therapy with high‐dose cytarabine for consolidation, as well as FLAG‐Ida and GO regimens were employed across these cases. Imatinib as maintenance was used in 10 (38.5%) patients, of which six (66.7%) achieved CR. Dasatinib was used in four patients (15.3%), of which two (50%) had CR. This suggests that TKI therapy may be an effective adjuvant treatment in de novo AML. A few studies reported that the *BCR::ABL1* fusion transcript was no longer detected after maintenance with TKIs, suggesting that TKIs may contribute to the suppression of a secondary clone [5, 25].

In our case report, the patient was treated with 7+3 induction, cytarabine for consolidation, and azacytidine with dasatinib for maintenance. He did not receive GO, nor was he suitable for allo‐SCT. Complete morphologic remission with hematologic response was obtained by Day 33 and complete molecular remission by Day 49. Although he achieved rapid CR on this regimen, he experienced multiple complications related to dasatinib. This case poses a number of questions, including the optimal timing, combination, and duration of TKI therapy in the context of dual rearrangement de novo AML. Further, whether GO should be used concurrently with TKIs and the impact of allo‐SCT in the management of these rare cases remains uncertain. Given the small number of cases reported, it is difficult to draw conclusions on the optimal therapy for de novo AML *BCR::ABL1* and *CBFB::MYH11* rearrangement. However, it appears that this patient subgroup may benefit from intensive chemotherapy regimens with the addition of TKI and GO. To date, there are no guidelines on how to manage this particular subtype of AML, which underscores the need to continue reporting cases in the literature.

## AUTHOR CONTRIBUTIONS

Brittany Salter wrote the manuscript and literature review; Sarah Ge conducted the literature review and developed the tables; Amy Tam and Dina Khalaf reviewed the manuscript and were involved in patient management; Suzanne Demczuk, Darci Butcher, and Elizabeth McCready carried out all the laboratory tests and were involved in patient management.

## CONFLICT OF INTEREST STATEMENT

The authors declare no conflict of interest.

## FUNDING INFORMATION

None.

## ETHICS STATEMENT

The authors have confirmed ethical approval statement is not needed for this submission.

## PATIENT CONSENT STATEMENT

Patient consent was provided by the patient (both verbal and written).

## CLINICAL TRIAL REGISTRATION

The authors have confirmed clinical trial registration is not needed for this submission.

## Data Availability

None.
